# Closed‐Loop Recyclable Polyhexahydrotriazine Aerogels Utilizing *N,N*‐Dimethyl Lactamide as a Green Solvent

**DOI:** 10.1002/cssc.202500125

**Published:** 2025-06-03

**Authors:** Chang‐Lin Wang, Ivona Glišić, Yi‐Ru Chen, Željko Tomović

**Affiliations:** ^1^ Department Polymer Performance Materials Group Department of Chemical Engineering and Chemistry Institute for Complex Molecular Systems (ICMS) Eindhoven University of Technology 5600 MB Eindhoven The Netherlands

**Keywords:** aerogel, green solvent, hexahydrotriazine, recycling, thermal insulation

## Abstract

Organic aerogels, known for their lightweight, highly porous structure, and ultralow thermal conductivity, have shown great potential in thermal insulation, chemical absorption, and energy storage. However, most aerogels to date pose environmental concerns, as their permanently cross‐linked scaffold makes recycling back to the original monomers virtually impossible. Additionally, the use of toxic solvents in aerogel fabrication raises further environmental and health concerns, challenging their sustainable application. Moreover, the development of next‐generation organic aerogels requires the design of recyclable materials with improved mechanical properties. In response to these challenges, this study demonstrates the synthesis of chemically recyclable polyhexahydrotriazine (PHT) aerogels based on an amide containing aromatic diamine, utilizing *N*,*N*‐dimethyl lactamide, a nontoxic and label‐free solvent, as gelation medium. Hansen solubility parameters provide key insights into how solvent choice influences the morphology and properties of PHT aerogels. The resulting PHT aerogels exhibit low bulk density (≈63 mg cm^−3^), high porosity (≈96%), excellent thermal insulation properties (≈17 mWm^−1^ K^−1^), and enhanced mechanical performance, all while being closed‐loop recyclable. This work highlights the importance of solvent selection in tuning aerogel properties and demonstrates a green route for fabricating sustainable, high‐performance thermal insulating materials.

## Graphical abstract

The synthesis of chemically recyclable polyhexahydrotriazine (PHT) aerogels, using *N*,*N*‐dimethyl lactamide, a nontoxic, label‐free solvent, is presented. The PHT aerogels exhibit low bulk density, high porosity, excellent thermal insulation, pronounced thermal stability, and enhanced mechanical performance. Furthermore, PHT aerogels can be depolymerized under acidic conditions, enabling recovery and reuse of monomers to prepare fresh aerogels with nearly identical properties.

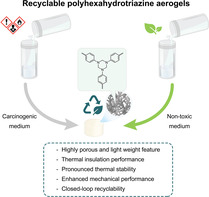

## 1 Introduction

Global energy consumption has reached its all‐time maximum, with forecasts predicting a further 14% increase by 2050.^[^ ^1^ ^]^ Notably, heating and cooling in the residential sector alone account for ≈48% of global building energy consumption, highlighting the urgent need for efficient thermal insulation technologies.^[^ ^2^ ^]^ As one of the top ten emerging technologies in chemistry, according to IUPAC, aerogels are regarded as a promising solution for effective energy conservation and the mitigation of excessive energy use.^[^ ^3^, ^4^ ^]^ The exceptional properties of organic aerogels (i.e., their low density <0.2 g cm^−3^, high specific surface area >300 m^2^ g^−1^, large porosity >85%, and ultralow thermal conductivity <20 mWm^−1^ K^−1^) make them superior to existing commercial insulation materials, such as polystyrene or polyurethane foams, and glass or rock wool.^[^ ^4^, ^5^ ^]^

Despite these advantages, organic aerogels, which typically consist of cross‐linked polymeric networks, are virtually nonrecyclable, raising serious environmental concerns regarding their end‐of‐life management. Thus, creating aerogels that combine exceptional properties with efficient recyclability presents a formidable challenge. To address this, it is essential to develop sustainable, recyclable aerogels, particularly with a focus on closed‐loop recycling. Previous research has used chemically reversible linkages, such as polyimine or polyhexahydrotriazine (PHT), to achieve the closed‐loop recycling of organic aerogels.^[^ ^6^, ^7^, ^8^, ^9^ ^]^ These studies demonstrated the recovery of monomers from the cross‐linked network through acid‐catalyzed depolymerization, enabling the regeneration of fresh aerogels. Taking it a step further, the mechanical robustness of recyclable aerogels can be enhanced by the incorporation of supramolecular interactions, such as hydrogen bonding, thereby broadening their range of applications.^[^ ^10^ ^]^

While previous studies address their sustainability by designing aerogels that can be recycled back to monomers, the use of carcinogenic, mutagenic, and reprotoxic solvents and chemicals during aerogel production still poses significant safety risks. In organic aerogel manufacturing, solvents play a crucial role as a medium for creating highly porous polymer network.^[^ ^5^, ^11^, ^12^, ^13^, ^14^, ^15^ ^]^ However, solvents that pose environmental and health hazards, such as chlorinated and aromatic solvents, continue to be widely used in aerogel production.^[^ ^16^, ^17^, ^18^ ^]^ According to one of the 12 principles of Green Chemistry: “Synthetic methods should be designed to use and generate substances that possess little or no toxicity to human health and the environment.”^[^ ^19^ ^]^ Hence, achieving the goals of environmental protection requires mass production using nontoxic, sustainable resources. Therefore, a sustainable approach to mitigate the environmental hazards associated with solvents is urgently needed.

**Figure 1:**
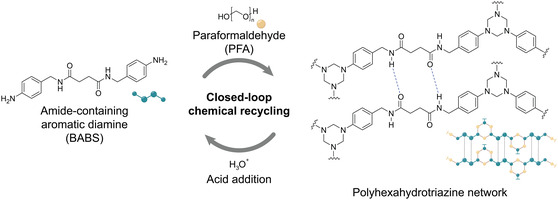
Circular flowchart of PHT network synthesis from the reaction between amide‐containing aromatic diamine (BABS) and paraformaldehyde (PFA) and the closed‐loop chemical recycling of such under acidic condition, recovering the starting monomers.

In this work, we introduce chemically recyclable organic aerogels based on PHT, prepared using *N,N*‐dimethyl lactamide (DML), a bio‐based, biodegradable, nontoxic solvent, as the gelation medium.^[^ ^20^, ^21^, ^22^ ^]^ To enhance the mechanical robustness of the PHT aerogels, we designed a new type of difunctional aromatic amine‐containing amide functional groups, which introduce hydrogen bonding during the sol–gel process (**Figure** **1**). As a result, the PHT aerogels synthesized in this study exhibit low bulk density (<0.07 g cm^−3^), high porosity (>95%), and a large specific surface area (>300 m^2^g^−1^). The PHT aerogel demonstrates ultralow thermal conductivity with value of 17.2 mWm^−1^ K^−1^, exhibiting excellent thermal insulation. These properties were found to be critically dependent on the gelation solvents, which prompted a systematic investigation into the effects of solvent choice and solvent blends on aerogel properties. Specifically, we used Hansen solubility parameters (HSPs) to rationalize the effect of solvents on the microstructural morphology and related thermal insulation performance. Moreover, closed‐loop recycling of PHT aerogels was achieved by applying acidic aqueous conditions to induce the selective cleavage of methylene moieties within the PHT network. The high recovery of pure amine monomers allows for the preparation of fresh aerogels with nearly identical properties to the original ones. Additionally, the solvents that were utilized for aerogel production can also be recovered with high yield and purity. Our approach to generating new closed‐loop recyclable, high‐performance aerogels through the use of green solvent contributes to the advancement of sustainable, thermally superinsulating materials.

## 2 Results and Discussion

### 2.1 Design and Fabrication of PHT Aerogels

**Figure 2:**
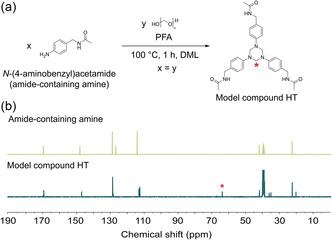
a) Reaction scheme of the model study on the formation of HT using equimolar ratio of *N*‐(4‐aminobenzyl)acetamide (amide‐containing amine) and PFA in DML at 100 °C for 1 h. b) ^13^C NMR spectra comparing the amide‐containing amine and model compound HT (100 MHz, 25 °C, DMSO‐*d*_6_).

The synthesis of the PHT network involves the trimerization reaction between aromatic amines and paraformaldehyde (PFA).^[^ ^23^, ^24^ ^]^ With the intention to create mechanically robust PHT network, we prepared a difunctional aromatic amine precursor with amide functional groups, namely *N*,*N*‐bis(4‐aminobenzyl)succinamide (BABS), for aerogel synthesis (Figure 1). Previous work showed that the exclusive formation of HT structure was achieved using equimolar amounts of amine and PFA.^[^ ^9^ ^]^ To gain further understanding of HT formation in the presence of amide moieties in DML as a solvent, a model reaction with *N*‐(4‐aminobenzyl)acetamide as a mono‐functional analog of BABS and PFA in equimolar ratio was performed. The reaction between two precursors was conducted in DML at 100 °C for 1 h (**Figure** **2**a). The reaction was monitored using NMR spectroscopy. New signals were observed at 4.8 ppm in the ^1^H NMR spectra and at 63 ppm in the ^13^C NMR spectra, indicating the formation of HT structures (Figure 2b and Figure S1, Supporting Information). Importantly, the sole formation of HT structures could be achieved without the presence of unwanted hemiaminal intermediate structures, which could lower the thermal stability and mechanical performance of the final materials.^[^ ^9^, ^25^ ^]^

**Figure 3:**
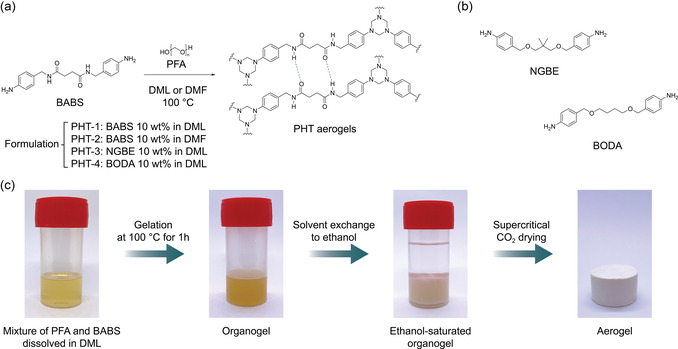
a) Reaction scheme of PHT formation. b) Molecular structure of other amines included in this chapter. c) Photographs of the synthesis of PHT‐1 aerogel.

Based on this knowledge, we employed the same reaction conditions for the aerogel synthesis based on aromatic diamine (BABS). To mitigate the potential environmental hazard of the organic solvents used for aerogel synthesis, DML was chosen as a sustainable solvent candidate. DML is produced on an industrial scale from lactic acid, a bio‐based resource,^[^ ^21^, ^26^ ^]^ and has been reported as a nontoxic and biodegradable solvent, making it an excellent alternative to other high‐boiling‐point organic solvents, such as DMF, DMAc, DMSO, and NMP.^[^ ^20^, ^22^, ^27^, ^28^ ^]^ In light of this, we prepared the PHT‐1 using DML as gelation solvent and utilized BABS as amine precursor to enhance its mechanical robustness (**Figure** **3**a). To compare the effect of solvent on the structure and properties of PHT aerogels, we also prepared PHT aerogel based on BABS using DMF as gelation solvent, namely PHT‐2. Furthermore, to compare the effect of amide functional groups present in BABS on the mechanical performance of aerogels, we also prepared two different sets of PHT aerogels using difunctional aromatic amines, 4,4′‐((2,2‐dimethylpropane‐1,3‐diyl)bis(oxy))dianiline (NGBE) and 4‐[4‐(4‐aminophenoxy)butoxy]aniline (BODA) without amide moieties, termed PHT‐3 and PHT‐4, respectively (Figure 3b).

The fabrication scheme of PHT aerogels is shown in Figure 3c. Initially, the amine monomers and PFA were separately dissolved in the selected solvent at 100 °C, maintaining a 1:1 molar ratio between the amine groups and formaldehyde. The trimerization reaction was initiated by combining the solutions at 100 °C, resulting in the formation of a stable organogel after 1 h. The solvent of the organogel was subsequently exchanged with ethanol, performed three times, each for 24 h, to completely remove the residual solvent, DML, used during the gelling step. Afterward, supercritical CO_2_ drying was conducted to remove the ethanol from the organogels without disrupting their delicate polymer structure, yielding PHT aerogels.

### 2.2 Chemical and Physical Properties of PHT Aerogels

**Figure 4:**
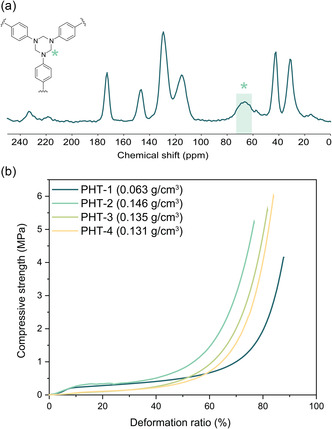
a) MAS ^13^C NMR spectrum of PHT‐1 aerogel. b) Stress‐deformation curves of PHT aerogels with the bulk density values of aerogels prepared in the size of 25 mm diameter and 15 mm height (Table S2, Supporting Information).

The chemical structure of PHT aerogels was investigated using MAS ^13^C NMR spectroscopy (**Figure** **4**a). According to the spectrum, the PHT‐1 aerogel shows the characteristic signal of HT around 65 ppm, indicating the methylene carbon of the HT‐core structure. Importantly, the absence of the hemiaminal structure verifies that the HT formation was quantitative without any side product formation, in agreement with the previous model study (Figure 2a).

The physical properties of PHT aerogels, including bulk density, linear shrinkage, and porosity, are summarized in **Table** **1**. All samples exhibited minimal shrinkage down to 3%, resulting in low bulk density. Notably, it was found that PHT‐1 had slightly lower conversion during synthesis. The unreacted precursors were washed out during the solvent‐exchange procedure, which resulted in the lowest bulk density of 63 mg cm^−3^. Nevertheless, PHT‐1 exhibits excellent mechanical stability and was used for further characterization. The porosity of the PHT aerogels, measured using a helium pycnometer, revealed that all aerogels exhibited porosity higher than 86%. In particular, PHT‐1 had the highest porosity of 96%, which was attributed to its low linear shrinkage (3%) and bulk density (63 mg cm^−3^).

**Table 1:** Material properties of PHT aerogels.

Name	Bulk density *ρ*_b_ [mg cm^−3^]	Linear shrinkage [%]a)	Skeletal density *ρ*_s_ [g cm^−3^]	Porosity Π [%]b)
PHT‐1	63	3	1.46	96
PHT‐2	170	16	1.26	86
PHT‐3	130	11	1.22	89
PHT‐4	130	8	1.25	90

a) Linear shrinkage was calculated based on the diameter change of the sample.

b) Porosity was calculated via equation: *Π* = (1 − *ρ*_b_/*ρ*_s_) × 100%.

Furthermore, the mechanical properties of the PHT aerogels were characterized by uniaxial compression tests (Figure 4b). According to the stress‐deformation curves, the mechanical performance varied depending on the monomers and solvents used during their preparation. Among the samples, those synthesized with BABS exhibited higher compressive modulus values, ranging from 1.1 to 1.6 MPa, whereas those prepared from other amines showed a modulus of around 0.2 MPa. A similar trend was also observed in the compressive strength of the aerogels, where PHT‐1 and PHT‐2 demonstrated higher compressive strengths, up to 266 kPa, while PHT‐3 and PHT‐4 exhibited much lower values, ranging from 52 to 62 kPa. This difference in mechanical performance could be attributed to the presence of amide moieties within the aerogel network, which forms supramolecular hydrogen bonds between different polymer chains, thereby physically enhancing the crosslink density and contributing to more robust structures than other types of organic aerogels (Figure S2a, Supporting Information). When comparing PHT‐1 and PHT‐2, PHT‐1 exhibited superior mechanical performance, with higher compressive and specific moduli (**Table** **2**). The enhanced mechanical performance of PHT‐1 could be attributed to the distinct nanoscale architectures of PHT‐1, as compared to PHT‐2, which are prepared from different solvent media, leading to variations in their mechanical robustness.

**Table 2:** Mechanical properties of PHT aerogels.

Name	Compressive modulus [MPa]a)	Specific modulus [m^−1^s^−1^]b)	Compressive strength [kPa]c)
PHT‐1	1.56 ± 0.36	0.022 ± 5E−3	226 ± 45
PHT‐2	1.09 ± 0.22	0.008 ± 2E−4	265 ± 44
PHT‐3	0.24 ± 0.07	0.002 ± 4E−4	52 ± 9
PHT‐4	0.18 ± 0.05	0.001 ± 3E−4	62 ± 1

a) Compressive modulus was calculated from the stress‐deformation curves obtained using sample of 25 mm diameter and 15 mm height.

b) Specific modulus was determined based on the ratio of compressive modulus and *ρ*_b_ of the sample sizes of 25 mm diameter and 15 mm height (Table S2, Supporting Information).

c) Compressive strength at 10% deformation ratio.

### 2.3 Microstructural Properties and Morphology of PHT Aerogels

**Figure 5:**
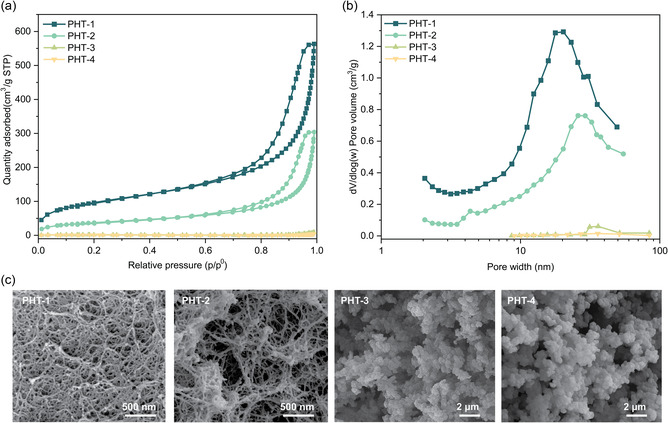
a) Nitrogen physisorption isotherm of PHT aerogels. b) Pore size distribution of PHT aerogels. c) SEM images of PHT aerogels.

The microstructural properties and morphology of PHT aerogels were investigated using nitrogen sorption porosimetry. According to the physisorption isotherms, PHT‐1 and PHT‐2 exhibit significantly higher nitrogen adsorption and desorption values compared to other PHT aerogels, suggesting that the use of BABS as monomers leads to more intricate nanostructural morphologies. (**Figure** **5**a). In addition, PHT‐1 also shows a much higher specific surface area (>340 m^2^g^−1^) when compared with the other PHT aerogels, indicating the abundance of its nanoscale polymer skeletons (**Table** **3**). Furthermore, the pore size distribution reveals that PHT‐1 and PHT‐2 have a broad range of pore sizes from 10 to 100 nm, whereas PHT‐3 and PHT‐4 exhibit only a limited number of pores smaller than 200 nm (Figure 5b). This suggests that the pores in PHT‐3 and PHT‐4 may be too large to be accurately measured by nitrogen physisorption.

**Table 3:** Microstructural properties of PHT aerogels.

Name	Specific surface area [m^2^ g^−1^]a)	Pore volume [cm^3^ g^−1^]b)
PHT‐1	344.9	0.90
PHT‐2	132.6	0.49
PHT‐3	4.8	0.01
PHT‐4	2.3	0.01

a) Calculated based on BET theory.

b) Calculated using BJH method.

To further investigate this phenomenon and study their overall surface morphologies, the PHT aerogels were examined using scanning electron microscopy (SEM). According to the SEM images, PHT‐1 and PHT‐2 display a fiber‐like topology, while PHT‐3 and PHT‐4 exhibit a cauliflower‐like morphology with much larger particle sizes (Figure 5c and Figure S3, Supporting Information). These observations align with the nitrogen physisorption results, where the specific surface area of PHT‐1 and PHT‐2 is significantly higher than the others. It also suggests that the amine precursors without the amide functional groups (NGBE and BODA) do not form intricate nanostructure as BABS. While both PHT‐1 and PHT‐2 show nanoscale fiber‐like morphology, the differences in specific surface area and pore volume are significant. This could arise from the large polymer clusters formed within PHT‐2 as shown in the SEM figures. We suspect that the formation of polymer clusters is due to the usage of DMF as the gelation solvent, which results in a microstructure distinct from that of PHT‐1. The anisotropic feature of PHT‐2 could also explain its poorer mechanical properties compared to PHT‐1.

**Figure 6:**
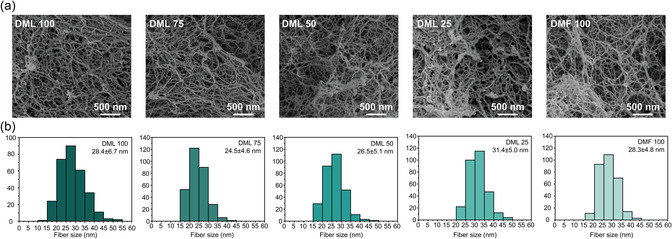
a) SEM images of PHT aerogels prepared from different solvent blends. b) Average fiber size histograms of the PHT aerogels prepared from different solvent blends.

To further study the solvent effect on surface morphology of aerogels, in addition to PHT‐1 and PHT‐2, we prepared PHT aerogels based on BABS monomers in the solvent blends of DML and DMF. The weight ratio of DML and DMF used was set at 100/0, 75/25, 50/50, 25/75, and 0/100; the specimen was named DML 100, DML 75, DML 50, DML 25, and DMF 100, respectively (Table S3, Supporting Information). Based on different compositions of the solvent blends, the respective HSPs were calculated to investigate their impact on materials properties (Table S4, Supporting Information). The bulk density and porosity of the PHT aerogels increased with increasing fraction of DMF. This behavior can be explained by the linear shrinkage of the samples, which could lead to high density. Regarding their pore structures, the aerogel prepared with higher content of DMF showed lower specific surface area and pore volume (Table S5, Supporting Information). The transition behavior could be correlated with the HSPs, where DMF shows lower hydrogen bonding parameter (δ_H_) of 11.3, whereas DML exhibits higher δ_H_ value of 15.9.^[^ ^28^, ^29^ ^]^ On the other hand, the changes in polarity (δ_p_) and dispersion forces (δ_d_) upon the addition of DMF were less significant compared to changes in hydrogen bonding capability. According to the SEM images, all PHT aerogels prepared in solvent blends exhibit a fiber‐like morphology. Their interconnected network is formed by fiber sizes ranging from 24 to 31 nm, which does not show significant difference (**Figure** **6** and Figure S4, Supporting Information). However, SEM images of DML 100 appear with isotropic distribution with uniform nanoscale fibers, in agreement with the appearance of PHT‐1. Conversely, increasing the content of DMF as a gelation solvent leads to an anisotropic morphology characterized by more pronounced polymer cluster formation and larger complementary pores (Figure S5−S7, Supporting Information). The cluster formation could be correlated with the lower hydrogen bonding capability of DMF, which results in pronounced polymer aggregation. These polymer clusters and associated anisotropic structural morphology result in larger complementary pore size giving rise to lower specific surface area and pore volume.

### 2.4 Thermal Properties of PHT Aerogels

**Figure 7:**
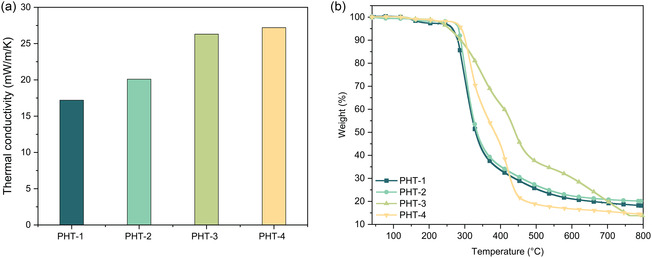
a) Thermal conductivity of PHT aerogels. b) TGA curves of PHT aerogels ranging from 40 to 793 °C with ramp rate of 10 °C min^−1^.

PHT aerogels, particularly PHT‐1, are expected to exhibit exceptional thermal insulation performance due to their intricate nanoscale structure. To demonstrate this, we measured the thermal conductivity of these aerogels using a heat flow meter according to the ASTM C518 standard (**Table** **4**). PHT‐1 showed the lowest thermal conductivity, with a value of 17.2 mWm^−1^ K^−1^, significantly lower than that of other aerogel materials (**Figure** **7**a and Figure S2b, Supporting Information). In contrast, PHT‐2 showed a higher thermal conductivity of 20.1 mWm^−1^ K^−1^, while PHT‐3 and PHT‐4 displayed even poorer insulation properties, with values ranging between 26 and 27 mWm^−1^ K^−1^. The difference in thermal insulation performance can be attributed to the composition and structure of the materials, which influence their thermal conductivities.

**Table 4:** Thermal properties of PHT aerogels.

Name	Thermal conductivity [mWm^−1^ K^−1^]	*T*_d5%_ [°C]a)	*T*_d30%_ [°C]b)	R_793_ [%]c)
PHT‐1	17.2 ± 0.04	265	304	18
PHT‐2	20.1 ± 0.02	245	308	20
PHT‐3	26.3 ± 0.04	254	366	14
PHT‐4	27.2 ± 0.02	290	330	14

a) Decomposition temperatures at 5% weight loss.

b) Decomposition temperatures at 30% weight loss.

c) Char residue at 793 °C.

In theory, heat transport in materials consists of three components: solid conduction, gas conduction, and radiation conduction.^[^ ^30^, ^31^ ^]^ For this analysis, the radiation component was neglected, leaving solid and gas conduction as the primary factors. To minimize thermal conductivity, reducing the solid conduction component is crucial, which can be achieved by lowering the bulk density of the material. PHT‐1 exhibited the lowest bulk density, while PHT‐2 and the other aerogels had higher densities, contributing to their increased solid conduction and overall higher thermal conductivity. Another method to reduce heat conduction is by minimizing gas conduction. Although the gas conductivity of air is known to be 26.7 mWm^−1^ K^−1^, it can be reduced by utilizing the Knudsen effect.^[^ ^30^, ^31^ ^]^ By designing a nanoscale microstructure with a pore size smaller than the mean free path of gas molecules, heat conduction can be further hindered. PHT‐1, with its isotropic nanoscale morphology, high specific surface area, and large pore volume, results in lower gas conductivity contributed by enhanced Knudsen effect. In contrast, PHT‐3 and PHT‐4, which exhibited very lower specific surface area, experienced no Knudsen effect, resulting in their higher thermal conductivities. In summary, the combination of reduced bulk density and intricate nanoarchitecture of PHT‐1 contributed to low thermal conductivity and thereby superior thermal insulation performance relative to the other aerogels.

Given that our PHT aerogels have a high content of aromatic and HT moieties, we can anticipate excellent thermal resistance from these materials.^[^ ^9^, ^32^, ^33^ ^]^ To evaluate their thermal stability, we conducted thermogravimetric analysis (TGA, Table 4 and Figure 7b). The PHT aerogels exhibited high *T*_d5%_ values, all exceeding 250 °C. These results also confirmed the successful formation of the HT structure, with no unwanted intermediates, specifically hemiaminals. It was found that hemiaminals have a significantly lower decomposition temperature of 200 °C, which can greatly reduce the thermal stability of PHT aerogels.^[^ ^24^, ^26^ ^]^ Therefore, the absence of hemiaminals in our PHT aerogels plays a pivotal role in maintaining their pronounced thermal stability.

### 2.5 Closed‐Loop Chemical Recycling of PHT Aerogel

**Figure 8:**
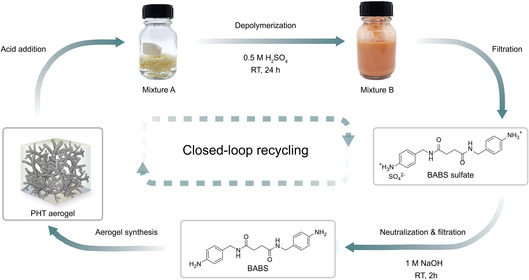
a) Schematic representative showing the protocol of closed‐loop recycling of PHT aerogel. PHT‐1 was added to 0.5 m H_2_SO_4_ at room temperature (recycled mixture A). And, the mixture was stirred at room temperature for 24 h, yielding recycled mixture B. Recycled mixture B was then filtered and BABS sulfate can be obtained. After neutralizing BABS sulfate with 1 m NaOH(aq) at room temperature for 2 h. The recycled BABS can be retrieved.

PHT aerogels are expected to enable efficient on‐demand depolymerization back to the monomers, as the incorporated HT moieties are known to hydrolyze when treated under acidic conditions (**Figure** **8**).^[^ ^24^, ^25^ ^]^ To illustrate the chemical recycling of our PHT aerogels, PHT‐1 was ground into small pieces and exposed to an aqueous 0.5 m H_2_SO_4_ solution at room temperature for 24 h. Under these conditions, the HT rings hydrolyzed into amine and formaldehyde, and the polymeric networks broke down in a quantitative and selective fashion. The formaldehyde was released as a gas, but could be easily recovered through distillation when the reaction is performed on an industrial scale.^[^ ^34^, ^35^ ^]^ On the other hand, the amine monomer (BABS) precipitated in the aqueous mixture as a sulfate salt. Filtration of the precipitates allowed convenient recovery of the BABS in its salt form (Figure 8). The BABS sulfate was then neutralized with 1 m NaOH_(aq)_ to yield neutral amine.

**Figure 9:**
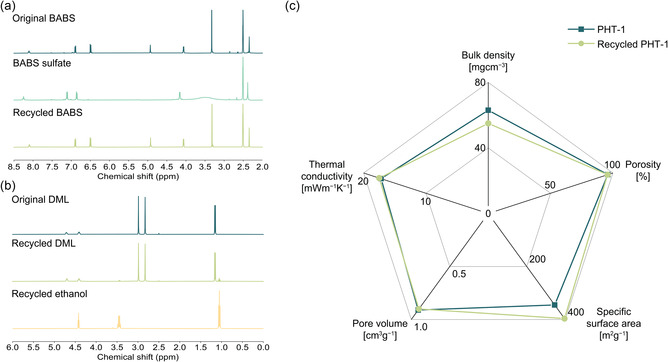
a) ^1^H NMR spectra of the original BABS, BABS sulfate, and recycled BABS (400 MHz, 25 °C, DMSO‐*d*_*6*_). b) ^1^H NMR spectra of the original DML, recycled DML, and recycled ethanol (400 MHz, 25 °C, DMSO‐*d*_*6*_). c) Radial graph comparing the aerogel‐specific properties of original and recycled PHT‐1s.

The purity of recycled BABS was estimated to be >99% by ^1^H NMR spectroscopy with recovery yield of up to 77% (**Figure** **9**a and Figure S8 and Table S6, Supporting Information). Since the isolated and virgin‐like monomers are ready for immediate reuse, the recycling loop is fully closed. In addition to recycling monomer precursors, we also demonstrated the potential of solvent recycling. By distillation of the solvent mixtures from the solvent‐exchange step, we achieved an ethanol recovery yield of up to 83% with high purity. Additionally, DML can be efficiently recovered through vacuum distillation at 110 °C with a recovery yield of 94% (Figures 9b and Table S6, Supporting Information). Consequently, all feedstock materials utilized in the manufacturing process can be successfully recycled. Since the isolated BABS monomers were ready for reuse, we prepared a new generation of PHT‐1 (recycled PHT‐1) using the recycled BABS together with fresh PFA. According to Figure 9c and Table S7, Supporting Information, all the aerogel‐specific properties of recycled PHT‐1 are in line with those of the original PHT‐1 material with low bulk density (55 mg cm^−3^), high porosity (96%), large specific surface area (399 m^2^ g^−1^.) More importantly, the recycled PHT‐1 attained the same level of thermal insulation performance of 17.6 mWm^−1^ K^−1^, demonstrating excellent reproducibility as thermal insulation material. Additionally, recycled PHT‐1 showed identical nitrogen physisorption isotherm along with pore size distribution as those of PHT‐1 (Figure S9a,b, Supporting Information). Their similar nanoscale morphology was further reflected on SEM images, where both aerogels revealed fiber‐like architecture (Figure S9c, Supporting Information). Furthermore, the TGA curve of recycled PHT‐1 indicated a significant resemblance with the original one, indicating similar thermal stability (Figure S9d, Supporting Information). Overall, we confirmed the viability of the closed‐loop recycling of our PHT aerogels by successfully fabricating fresh aerogel from recycled monomers with nearly identical properties.

## 3 Conclusion

In this work, we propose a new aerogel synthetic protocol that involves label‐free, nontoxic solvent medium to fabricate high‐performance PHT aerogel. Furthermore, PHT aerogel also showed full recyclability by recovering the monomers with high purity and yield. Besides the ultralow thermal conductivity (≈17 mWm^−1^ K^−1^), low bulk density (≈0.06 g cm^−3^), and high thermal stability (*T*_d5%_ > 250 °C), this material also exhibited enhanced mechanical performance due to the hydrogen bonding interaction between the amide moieties of the polymer network. We also found that the solvent used for the gelation step significantly influences the aerogel structure and performance. Through an investigation of DML/DMF solvent blends, we demonstrated that HSPs are effective in elucidating the role of solvent in shaping the nanostructure of PHT aerogels. Overall, this work introduces a promising strategy for developing high‐performance recyclable aerogels, where the synergy of hydrogen bonding‐induced mechanical enhancement, green solvent utilization, and full recyclability collectively represents a key green chemistry practice that has yet to be fully realized. This work serves as a strong foundation for future studies aimed at advancing sustainable aerogel materials for various applications.

## 4 Experimental Section

4.1

4.1.1

##### Materials

4‐(Aminomethyl)aniline, 4,4′‐((2,2‐dimethylpropane‐1,3‐diyl)bis(oxy))dianiline (NGBE), and 4‐[4‐(4‐aminophenoxy)butoxy]aniline (BODA) were purchased from BLD Pharm. B.V. Sulfuric acid (98%), sodium hydroxide, and paraformaldehyde (PFA) were purchased from Merck Life Science B.V. Dimethyl succinate was purchased from TCI Europe B.V. Lanthanum trifluoromethanesulfonate (La(OTf)3, 99%) was purchased from Alfa Aesar Ltd. Dimethyl formamide (DMF), acetone, ethyl acetate, methanol, dichloromethane, and ethanol were purchased from Biosolve B.V. N,N‐Dimethyl lactamide (DML) as commercial product Agnique AMD 3 L was provided by BASF SE, Germany. DMSO‐*d*_6_ (99.9% D) obtained from Cambridge Isotope Laboratories. *N*‐(4‐aminobenzyl)acetamide was synthesized according to previously published literature (See Supporting Information for more details).^[^ ^36^ ^]^ Liquid CO_2_ (grade 2.7), nitrogen (grade 5.0), and helium (grade 4.6) were purchased from Linde gas B.V.

##### Characterization

For the characterization of aerogels, PHT aerogels with sample dimensions of 55 mm diameter and 10 mm thickness were used unless mentioned otherwise.

The chemical structures of original and recycled BABS were identified by nuclear magnetic resonance (NMR) spectroscopy conducted on a Bruker UltraShield spectrometer (400 MHz for ^1^H NMR and 100 MHz for ^13^C NMR) at 25 °C with DMSO‐*d*_6_ as solvent. The chemical structures of PHT aerogels were analyzed using an 11.7 T Bruker NMR spectrometer operating at 125 MHz for ^13^C NMR spectra. ^13^C MAS NMR experiments were performed using a Bruker triple channel 4 mm MAS probe head spinning at 13 kHz. ^13^C MAS NMR spectra were recorded using a ^13^C cross‐polarization (CP) pulse sequence with a ramped contact pulse of 1 ms and an interscan delay of 3 s. NMR chemical shift calibrations of ^13^C NMR spectra were performed using solid adamantane as a reference.

Mass spectroscopy analysis was performed with an Autoflex maX, Bruker‐MALDI‐TOF Mass Spectrometer equipped with a 355 nm Nd: YAG smartbeam laser. α‐Cyano‐4‐hydrocycinnamic acid (CHCA) and 2‐[(2E)‐3‐(4‐tert‐butylphenyl)‐2‐methylprop2‐enylidene] malononitrile (DCTB) were used as matrices. The sample was solubilized in THF at a concentration of 2 mg mL^−1^.

The porosity of the PHT aerogels was studied by nitrogen physisorption porosimetry. The specific surface area and pore size distribution of the aerogels were analyzed by Brunauer–Emmett–Teller (BET) analyzer (TriStar II Plus). Before measurement, the samples were outgassed at 80 °C for 2 h under vacuum. Nitrogen (grade 5.0) and helium (grade 4.6) were chosen to measure physisorption isotherm. The porosity and skeletal density of the PHT aerogels were measured by helium pycnometer (AccuPyc II 1345) using helium grade 4.6. 10 data points were taken with 10 equilibrium cycles. The morphology of PHT aerogels was characterized by SEM (FEI Quanta 200 3D) at acceleration voltage of 10 kV. The aerogel samples were sputtered with gold for 40 s before testing.

The uniaxial compression test of PHT aerogels was conducted by ZwickRoell Materials Testing Machine, Zwicki Z2.5/TN. PHT aerogels with sample dimensions of 25 mm diameter and 15 mm thickness were used. The compressive modulus was calculated between 0.05% and 0.25% deformation ratio.

The thermal properties of PHT aerogels were assessed by TGA 550 (TA Instruments) under nitrogen atmosphere at the heating rate of 10 °C min^−1^ from 40 to 793 °C. The thermal conductivity of PHT aerogels was measured by heat flow meter (Thermtest Inc., HFM‐25) at 20 °C and 20−35% humidity according to ASTM C518 international standard. Prior to the measurement, the machine was calibrated with EPS 1450 E as reference material. The aerogel sample dimensions of 55 mm diameter and 10 mm thickness were used.

##### Synthesis of N,N‐bis(4‐aminobenzyl)succinamide (BABS)

Dimethyl succinate (14.98 g, 102.50 mmol, 1 eq.) and 4‐(aminomethyl)aniline (31.37 g, 256.77 mmol, 2.51 eq.) were placed in a 3‐neck flask equipped with a condenser. The mixture was stirred and heated to 80 °C under reflux for 48 h with Ar flow. After cooling down, the solid mixture was filtered and washed with acetone. Finally, the solid was dried in a vacuum oven at 50 °C for 3 h. The resulting white solid was obtained with a yield of 70%. ^1^H NMR (400 MHz, 25 °C, DMSO‐*d*_6_): δ = 8.10 (t, *J *= 5.8 Hz, 2 H), 6.89 (d, *J* = 8.3 Hz, 4 H), 6.49 (d, *J* = 8.4 Hz, 4 H), 4.93 (s, 4 H), 4.06 (d, *J* = 5.7, 4 H), 2.34 (s, 4 H); ^13^C NMR (161.9 MHz, 25 °C, DMSO‐*d*_6_): δ = 171.6, 147.9, 128.7, 126.9, 114.2, 42.4, 31.4 ppm. MS (m/z): [M + H]^+^ calc. for C_18_H_23_N_4_O_2_^+^, 327.17; found, 327.17.

##### Model Study of Hexahydrotriazine (HT) Formation in DML

*N*‐(4‐Aminobenzyl)acetamide (0.50 g, 3.04 mmol, 1 eq.) and PFA (0.09 g, 3.04 mmol, 1 eq.) were added to a 10 mL round‐bottom flask and the reagents were dissolved in DML (6.09 mL). The reaction mixture was stirred at 100 °C under N_2_ atmosphere for 1 h. Following the reaction, the solvent was removed under vacuum. The product was isolated as a yellow solid, obtained without further purification (0.50 g). ^1^H NMR (400 MHz, 25 °C, DMSO‐*d*_6_) δ = 8.21 (t, J = 5.5 Hz, 3H), 7.09 (d, *J* = 6.8 Hz, 6 H), 7.01 (d, *J* = 8.4 Hz, 6 H), 4.80 (s, 6 H), 4.12 (d, *J* = 5.2 Hz, 6 H), 1.83 (s, 9 H). ^13^C NMR (100 MHz, 25 °C, DMSO‐*d*_6_) 168.76, 146.52, 128.32, 127.94, 112.83, 112.29, 63.81, 41.90, 41.87, 22.60. MS (MALDI‐TOF) m/z calc. for C_30_H_35_N_6_O_3_ [M—H^−^] 527.28, found 527.27.

##### PHT Aerogels Preparation

Preparation of PHT‐1 is used as an example (Table S1, Supporting Information): The PHT organogel was prepared by mixing components A and B. Component A consisted of 2.62 g BABS dissolved in 13.5 g DML, while component B contained 0.48 g PFA dissolved in 13.5 g DML. Both components were prepared in a polypropylene (PP) vial by dissolving monomers at 100 °C. The gelation was initiated by mixing the two components into one vial, which was then shaken until a homogeneous solution was obtained. The solution was poured into PTFE mold with a diameter of 65 mm and was then placed at 100 °C for 1 h until gelation. Following gelation, the organogel was sealed and allowed to age for 24 h under ambient conditions. Subsequently, the organogel underwent solvent exchange with 200 mL of ethanol, repeated thrice, each for 24 h. The ethanol‐saturated organogel was then transferred into an autoclave, submerged in ethanol, and sealed in a supercritical fluid‐extraction autoclave. The pressure was maintained at 100 bar and the temperature was maintained above 60 °C with the constant inflow of CO_2_. The mixture of solvents and CO_2_ was vented out multiple times during the drying while withstanding the pressure and temperature. Subsequently, the aerogel was then stored in a vacuum oven at 80 °C for 24 h to ensure complete removal of the solvent. The dried sample was stored in a desiccator chamber with a relative humidity of 30%. The detailed composition of PHT aerogels is summarized in Table S1, Supporting Information.

##### Closed‐Loop Recycling of PHT Aerogel and Solvents

PHT‐1 (2.05 g) was added to 0.5 m H_2_SO_4_ (20 mL) in a 50 mL vial. The mixture was stirred at room temperature for 24 h. After complete depolymerization, the precipitated BABS sulfate was filtered and washed with distilled water. The BABS salt was then neutralized with 1 m aqueous NaOH (100 mL) solution at room temperature for 2 h, which led to the precipitation of BABS in its amine form. The precipitated BABS was filtered, washed with distilled water, dried in a vacuum oven at 80 °C overnight, and finally obtained as off‐white powder in 76% yield. Additionally, the solvents used during the solvent exchange procedure were collected for closed‐loop recycling. The solvent mixture was distilled at 80 °C to recover the ethanol with recovery yield of 83%. The residual solvent was subsequently distilled using vacuum distillation at 110 °C to recover the pure DML solvent with recovery yield of 94%.

## Conflict of Interest

The authors declare no conflict of interest.

## Supporting information

Supplementary Material

## Data Availability

The data that support the findings of this study are available from the corresponding author upon reasonable request.;
