# Supplementary material for: Closed‐Loop Recyclable Polyhexahydrotriazine Aerogels Utilizing N,N‐Dimethyl Lactamide as a Green Solvent
Source: ChemSusChem. 2025 Jun 3;18(14):e202500125. doi: 10.1002/cssc.202500125 (PMC12270357; doi:10.1002/cssc.202500125)
Supplement: Supplementary file 1 — Supplementary Material [file CSSC-18-e202500125-s001.pdf]

Supporting Information

Closed-Loop Recyclable Polyhexahydrotriazine Aerogels Utilizing N,N-Dimethyl Lactamide as a Green Solvent

Chang-Lin Wang,[a] Ivona Glišić,[a] Yi-Ru Chen,[a] and Željko Tomović*[a]

[a] Polymer Performance Materials Group, Department of Chemical Engineering and Chemistry and Institute for Complex Molecular Systems (ICMS), Eindhoven University of Technology, 5600 MB Eindhoven, The Netherlands.

Corresponding Author

Željko Tomović

E-mail: z.tomovic@tue.nl

Table of Contents

1. Model reaction 3

1.1 Synthesis of *N*-(4-aminobenzyl)acetamide 3

2. Preparation of aerogels 5

2.1 Supercritical CO_2 drying setup 5

2.2 Aerogel formulation tables.. 6

2.3 Comparison of aerogel properties 7

2.4 Morphology of PHT aerogels 8

3. PHT aerogels synthesis using different solvent blends.. 8

3.1 Hansen solubility calculation of different solvent blends 9

3.2 Investigation of the relationship between Hansen solubility parameters of solvent blends and aerogel properties 10

4. Closed-loop recycling of PHT aerogel and solvents.. 14

5. Supplementary figures 16

6. References.. 18

1. **Model reaction**

1.1 Synthesis of *N*-(4-aminobenzyl)acetamide[1]

In an oven-dried 10 mL round-bottom flask equipped with a stir bar, $La(OTf)_3$ (332.6 mg, 0.057 mmol) was added and the flask was flame-dried under vacuum then backfilled with argon for three times. Subsequently, 4-(Aminomethyl)aniline (1.66 g, 13.62 mmol) and ethyl acetate (1.11 mL, 11.35 mmol) were introduced to the flask. The mixture was stirred at 50 °C for 24 h. After monitoring the completion of reaction by TLC, the crude mixture was diluted with dichloromethane and purified by column chromatography eluting with ethyl acetate/methanol = 1/0 to 9/1 to afford the desired product as light-yellow oil (1.72 g, 92% yield). ^{1}H NMR (400 MHz, 25 °C. DMSO-d_6): δ = 8.15 (t, *J*=5.8 Hz, 1H), 6.92 (d, *J*=8.4 Hz, 2H), 6.53 (d, *J*=8.4 Hz, 2H), 4.94 (s, 2H), 4.08 (d, *J*=5.8 Hz, 2H), 1.84 (s, 3H). ^{13}C NMR (100 MHz, 25 °C, DMSO) δ = 169.08, 147.58, 128.45, 126.48, 113.85, 42.08, 22.68. MS (MALDI-TOF) m/z calc. for $C_9H_{12}N_2O$ [M^+] 164.09, found 164.18.

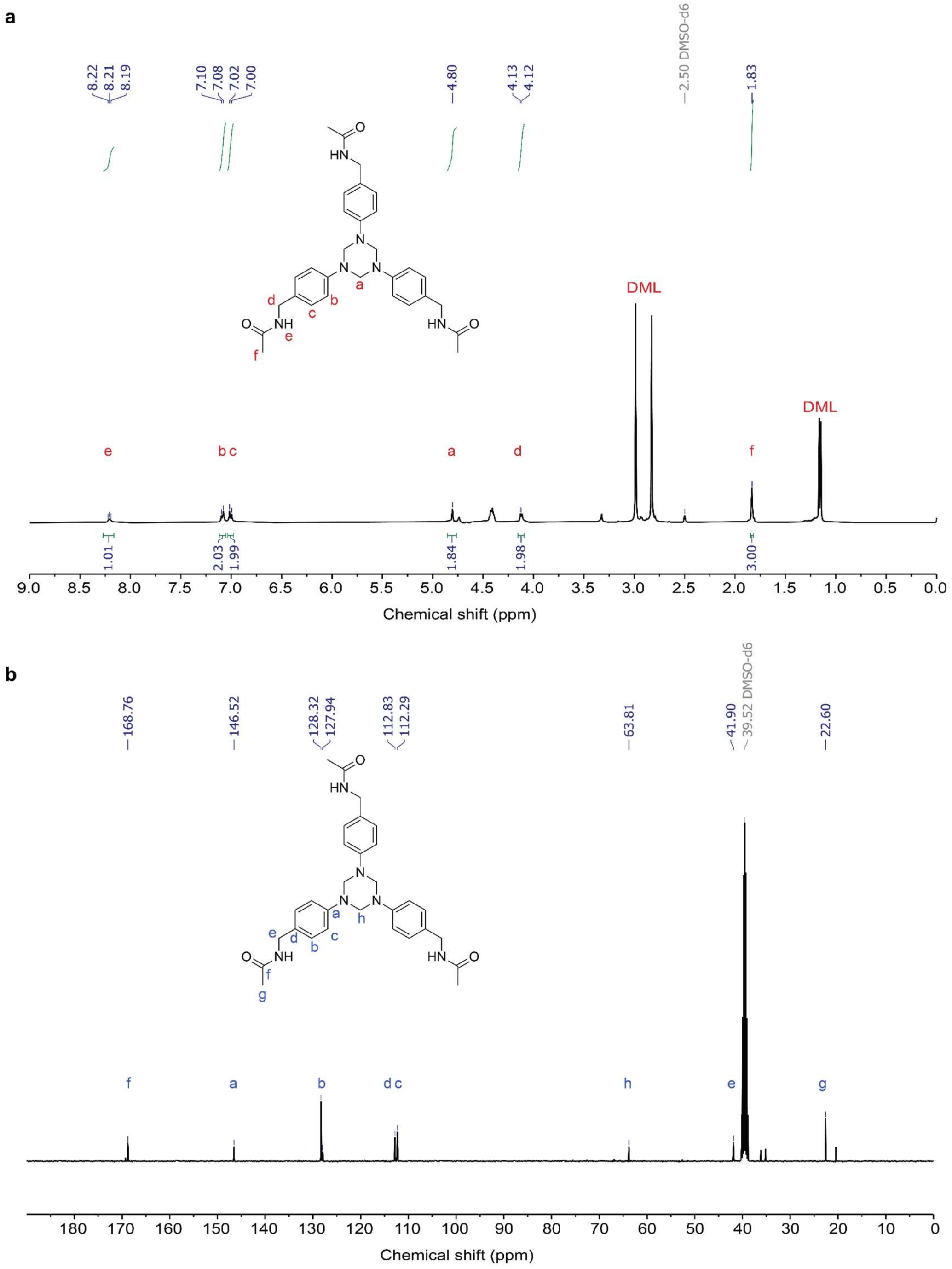

Figure S1. a) ^{1}H NMR, b) ^{13}C NMR spectra of the HT product from the model reaction between *N*-(4-aminobenzyl)acetamide and PFA (400 MHz, 25 °C, DMSO-d_6).

2. Preparation of aerogels

2.1 Supercritical CO_2 drying setup

Liquid CO_2 grade 2.7 (purity > 99.7%) is used as exchange agent for the supercritical drying process (SCD). The high pressure extraction/drying units "HP-DE200" is utilized as the drying setup. It comprises one autoclave (**Scheme S1**), provided by Eurotechnica, with a maximum working temperature of 100 °C and allowable operation pressure of 220 bar. The autoclave includes a thermowell with a NiCr-Ni thermocouple to measure the internal temperature during the process. Two venting tubes are also attached to the autoclave to extract the covering solvent and depressurization. Apart from the autoclave (9), the supercritical drying system employed in this work consists of two thermal baths (Selecta, UNITRONIC 200) for heat exchanger 1 and 2 (4 and 7), a mechanical pump (5) (provided by Maximator), a check number valve (2), 5 needle valve (3,6,7,8,10 and 11) and a CO_2 bottle (1) (**Scheme S1**).

Gels are first introduced into the autoclave (9) and covered with the solvent used for gel formation. This was done to avoid premature solvent evaporation that could lead to a higher shrinkage. Then, CO_2 is gradually pressurized up to 100 bar, extracting the solvent from the inside of the wet gel pores. The heat exchanger 2 (7) maintains the autoclave at constant temperature of 60 °C. Once supercritical conditions are achieved, the solvent is extracted from the gels in the autoclave (9) (**Scheme S1**). The supercritical CO_2 enriched with extracted solvent is vented out by releasing the autoclave. During this process, the pressure is maintained above with constant fresh CO_2 input. The venting process takes around 10 to 15 min and the autoclave will be closed to reach further extraction. Three cycles of extraction were further conducted with waiting interval of 30 min each. Finally, when the aerogel pores are completely free of solvent, pressure is slowly released to atmospheric pressure through the metering valve for 45 min.

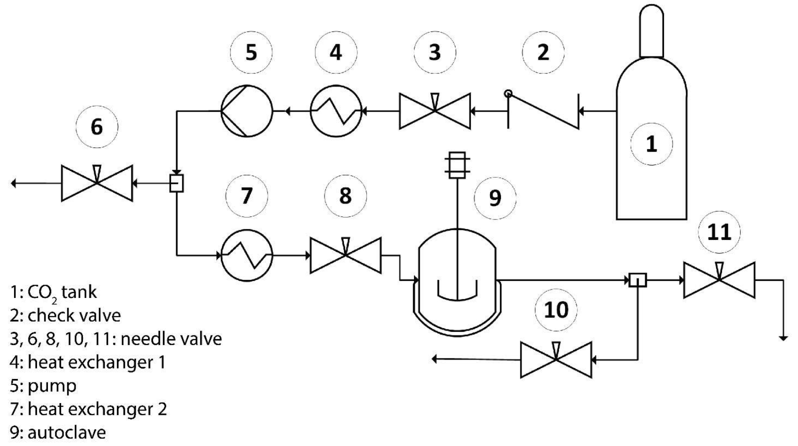

Scheme S1. Flow diagram of supercritical drying setup.

2.2 Aerogel formulation tables

Table S1. Formulation of PHT aerogels

Name	BABS [g]	NGBE [g]	BODA [g]	PFA [g]	DML [g]	DMF [g]
PHT-1	2.62	-	-	0.48	27	-
PHT-2	2.90	-	-	0.53	-	27
PHT-3	-	2.56	-	0.54	27	-
PHT-4	-	-	2.54	0.56	27	-
PHT-1[a)]	0.87	-	-	0.16	9	-
PHT-2[a)]	0.97	-	-	0.18	-	9
PHT-3[a)]	-	0.85	-	0.18	9	-
PHT-4[a)]	-	-	0.85	0.19	9	-

a) Samples were prepared for compression testing with the dimensions of 25 mm diameter and 15 mm height.

Table S2. Bulk density of PHT aerogels for compression testing

Name	Bulk density ρ_b [$mgcm^{-3}$]
PHT-1[a)]	70.5 ± 3.1
PHT-2[a)]	146.1 ± 3.7
PHT-3[a)]	135.0 ± 5.9
PHT-4[a)]	126.4 ± 3.3

a) Samples were prepared with the dimensions of 25 mm diameter and 15 mm height.

2.3 Comparison of aerogel properties

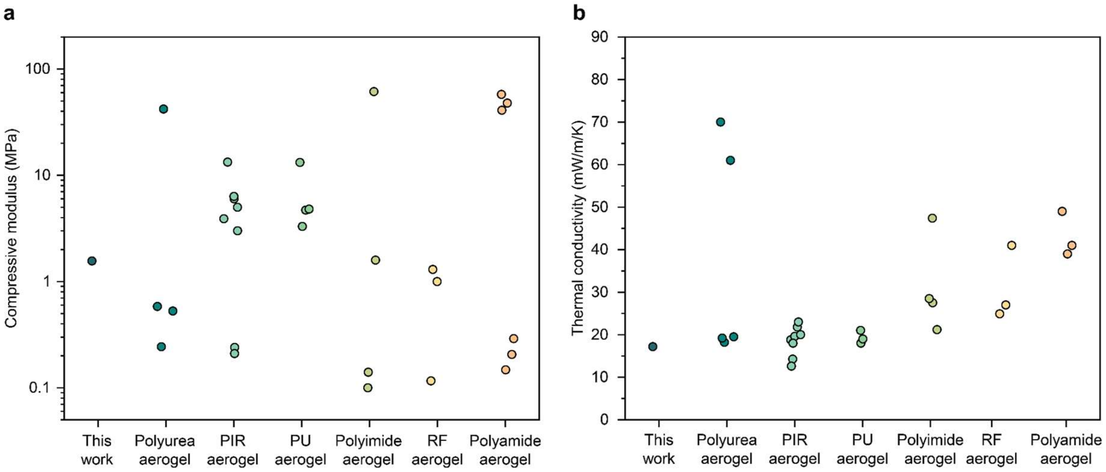

Figure S2. a) Comparison of compressive modulus of PHT-1 aerogel with other organic aerogels, including polyurea aerogels,[2,3] polyisocyanurate (PIR) aerogels,[4–6] polyurethane (PU) aerogels,[7,8] polyimide aerogels,[9–12] resorcinol-formaldehyde (RF) aerogels,[13,14] and polyamide aerogels.[15,16] It is important to notice that compressive modulus values are reported for various aerogel materials having different bulk densities. b) Comparison of thermal conductivity of PHT aerogels with other organic aerogels, including polyurea aerogels,[2,3] polyisocyanurate (PIR) aerogels,[4–6] polyurethane (PU) aerogels,[7,8] polyimide aerogels,[9–12] resorcinol-formaldehyde (RF) aerogels,[13,14] and polyamide aerogels.[15,16]

2.4 Morphology of PHT aerogels

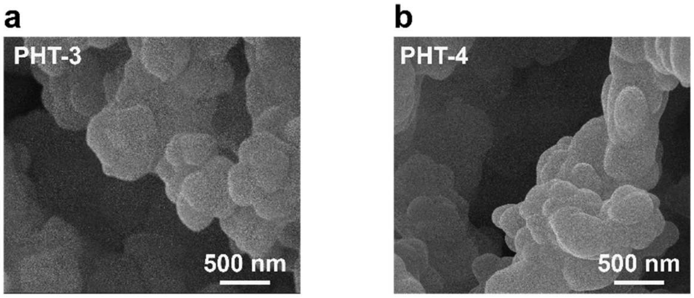

Figure S3. SEM images of PHT aerogels with 100,000 magnification. a) PHT-3, b) PHT-4.

3. PHT aerogels synthesis using different solvent blends

The series of solvent blends were prepared using DML and DMF. The solvent ratio of two solvents were set as: 100/0, 75/25, 50/50, 25/75, 0/100 weight fraction. The synthesis of the aerogels was followed by general PHT aerogel preparation described in the experimental section. The detailed composition of PHT aerogels is summarized in supporting information (**Table S3**).

Table S3. Formulation of PHT aerogels prepared using different solvent blends

Name	BABS [g]	PFA [g]	DMF [g]	DML [g]
DML 100	0.87	0.160	-	9.00
DML 75	0.90	0.165	2.25	6.75
DML 50	0.92	0.170	4.50	4.50
DML 25	0.94	0.175	6.75	2.25
DMF 100	0.96	0.180	9.00	-

3.1 Hansen solubility calculation of different solvent blends

Hansen solubility parameters of the solvent blends between DML and DMF were calculated using the following equations:[17]

$$\delta_{d,mixture} = \chi_{DMF}\delta_{d,DMF} + \chi_{DML}\delta_{d,DML} \quad \text{eq. 1}$$

$$\delta_{p,mixture} = \chi_{DMF}\delta_{p,DMF} + \chi_{DML}\delta_{p,DML} \quad \text{eq. 2}$$

$$\delta_{h,mixture} = \chi_{DMF}\delta_{h,DMF} + \chi_{DML}\delta_{h,DML} \quad \text{eq. 3}$$

Where δ_d is the energy from dispersion forces between molecules ($MPa^{1/2}$); δ_p is the energy from dipolar intermolecular forces between molecules ($MPa^{1/2}$); δ_h is the energy from hydrogen bonds between molecules ($MPa^{1/2}$). χ is the volume fraction of the solvent in the solvent blends. The solubility parameters of DML and DMF were taken from the reported literature.[18]

The volume fractions of the solvent (χ) were calculated based on the weight fractions and densities of each solvent using the following equations:

$$\chi_{DMF} = \frac{v_{DMF}}{v_{mixture}} \quad \text{eq. 4}$$

$$\chi_{DML} = \frac{v_{DML}}{v_{mixture}} \quad \text{eq. 5}$$

where v_{DMF} is the solvent volume of DMF, v_{DML} is the solvent volume of DML and $v_{mixture}$ is the solvent volume of the mixture.

The results of the calculations are summarized in **Table S4**.

Table S4. Hansen solubility parameters of solvent blends between DML and DMF

Name	m_{DMF} [a)]	m_{DML} [a)]	χ_{DMF}	χ_{DML}	δ_d [$MPa^{1/2}$]	δ_p [$MPa^{1/2}$]	δ_d [$MPa^{1/2}$]
DML 100	1.00	0.00	1.00	0.00	18.40	12.90	15.90
DML 75	0.75	0.25	0.77	0.23	18.12	13.11	14.65
DML 50	0.50	0.50	0.53	0.47	17.87	13.32	13.48
DML 25	0.25	0.75	0.27	0.73	17.62	13.51	12.36
DMF 100	0.00	1.00	0.00	1.00	17.40	13.70	11.30

a) m_{DMF} is the mass fraction of DMF and m_{DML} is the mass fraction of DML.

3.2 Investigation of the relationship between Hansen solubility parameters of solvent blends and aerogel properties

To further study the solvent effect, we prepared PHT aerogels in the solvent blends of DML and DMF based on BABS monomers. The weight ratio of DML and DMF used were set at 100/0, 75/25, 50/50, 25/75, and 0/100; the specimen was named as DML 100, DML 75, DML 50, DML 25, and DMF 100, respectively (**Table S3**). Based on different compositions of the solvent blends, the respective Hansen solubility parameters were calculated to investigate its impact on materials properties (**Table S4**).

The properties of PHT aerogels from different solvent blends display significant scatter when plotted against the Hansen solubility parameters (**Figure S6**). This scatter arises because the aerogel properties are not solely defined by a single solubility parameter. For example, solvent mixtures with a specific δ_D can vary significantly in their δ_P and δ_H values. Additionally, the Hansen solubility parameters reduce the complexity of a solvent mixture to three numerical descriptors, which inherently simplifies the chemical and physical interactions. Nonetheless, certain general trends can be observed.

PHT aerogels with lower bulk density were typically synthesized in solvents with higher δ_H values, showing that a high δ_H parameter is critical for achieving lightweight materials (**Figure S6a-1**). Similarly, linear shrinkage of the aerogels shows a strong dependence on δ_H, with shrinkage decreasing as δ_H rises, indicating that stronger intermolecular interactions promote structural stability (**Figure S6a-2**). Conversely, positive trends are observed for porosity, specific surface area, and pore volume, where higher δ_H value corresponds to more intricate nanomaterials, present with higher parameter value (**Figure 6a-3−5**). In contrast, all aerogel specific parameters show less dependence on δ_D and δ_P. It can be observed that the influence of δ_D and δ_P are less pronounced, as shown by the significant scatter in the properties data as a function of δ_D and δ_P (**Figure S6b** and **S6c**).

The observed trends highlight the dominant role of δ_H in defining the physical and structure properties of our PHT aerogels. It appears to dictate the optimal solvent conditions for achieving low-density, highly porous aerogels with desirable nanoscale structural morphology. Meanwhile, δ_D and δ_P play secondary roles in controlling the aerogel physical properties, as evidenced by the lack of clear trends in several property-parameter correlations.

Table S5. Material properties of PHT aerogels prepared using different solvent blends[a)]

Name	Bulk density [mgcm^{-3}]	Linear shrinkage [%]	Skeletal density [gcm^{-3}]	Porosity [%]	Specific surface area [m^{2}g^{-1}]	Pore volume [cm^{3}g^{-1}]	Fiber width [nm][b)]
DML 100	64 ± 4	2 ± 0.0	1.50 ± 0.002	96 ± 0.0	359 ± 37	1.06 ± 0.10	28.4 ± 6.7
DML 75	86 ± 10	9 ± 4.0	1.42 ± 0.016	95 ± 0.7	240 ± 28	0.70 ± 0.04	24.5 ± 4.6
DML 50	104 ± 4	10 ± 1.2	1.43 ± 0.031	93 ± 0.0	188 ± 26	0.55 ± 0.11	26.5 ± 5.1
DML 25	118 ± 2	12 ± 0.6	1.55 ± 0.189	93 ± 0.6	158 ± 10	0.48 ± 0.09	31.4 ± 5.0
DMF 100	145 ± 16	16 ± 2.1	1.39 ± 0.058	90 ± 0.7	136 ± 8	0.46 ± 0.06	28.3 ± 4.8

[a)]Two batches of the samples were prepared. [b)]300 points were taken using ImageJ software.

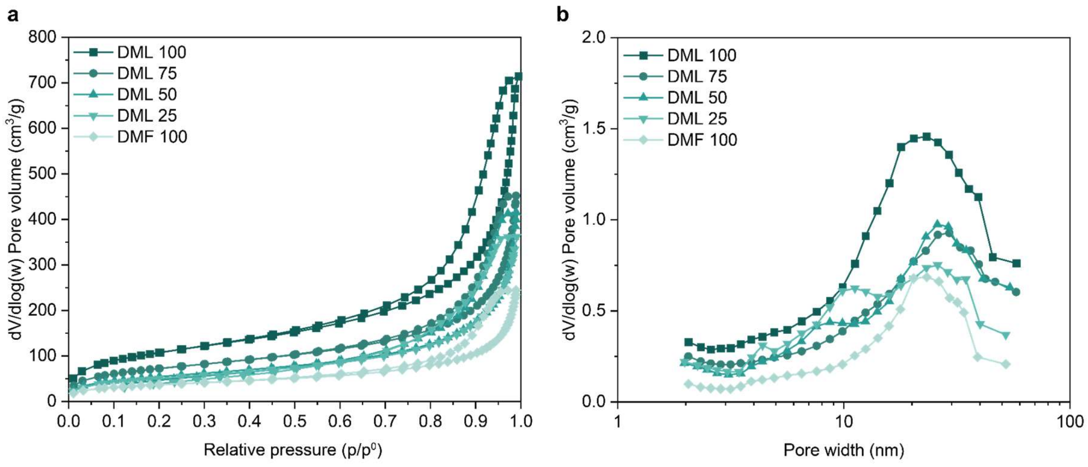

Figure S4. a) Nitrogen physisorption isotherm of PHT aerogels prepared from different solvent blends. b) Pore size distribution of PHT aerogels prepared from different solvent blends.

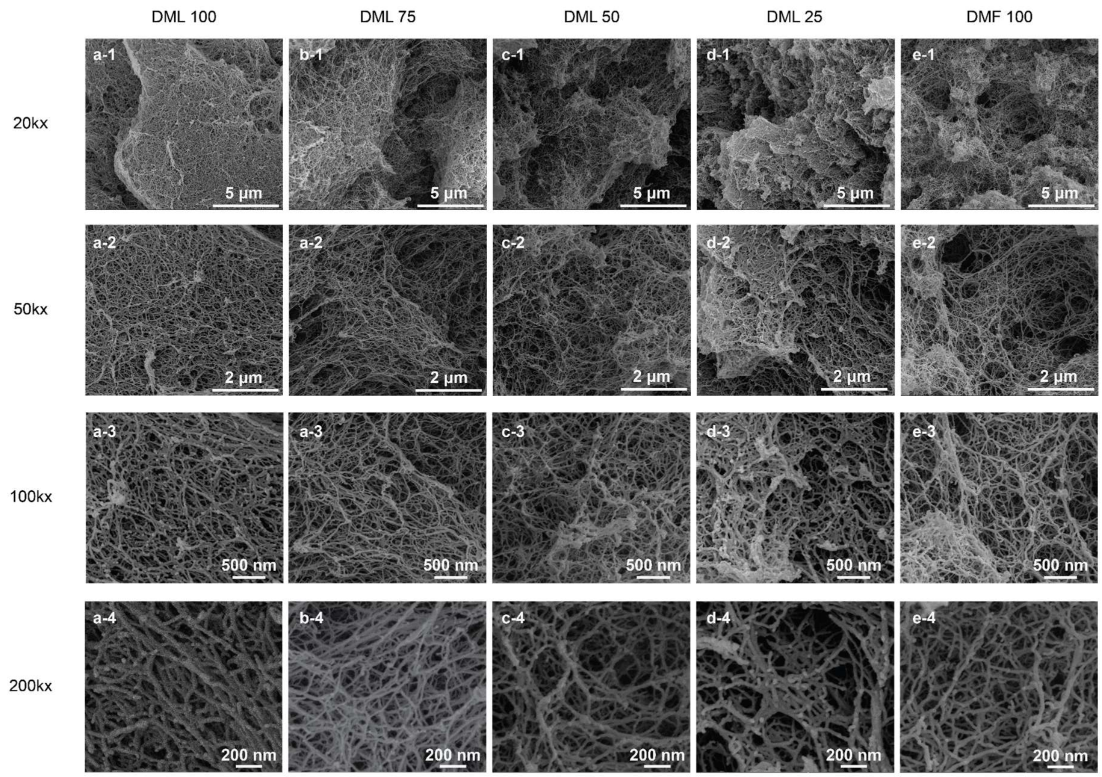

Figure S5. SEM images of PHT aerogels prepared using different solvent blends. DML100 (a-1−5): pure DML; DML 75 (b-1−5): DML/DMF (75/25 w/w); DML 50 (c-1−5): DML/DMF (50/50 w/w); DML 25 (d-1−5): DML/DMF (25/75 w/w) ; DMF 100 (e-1−5): pure DMF.

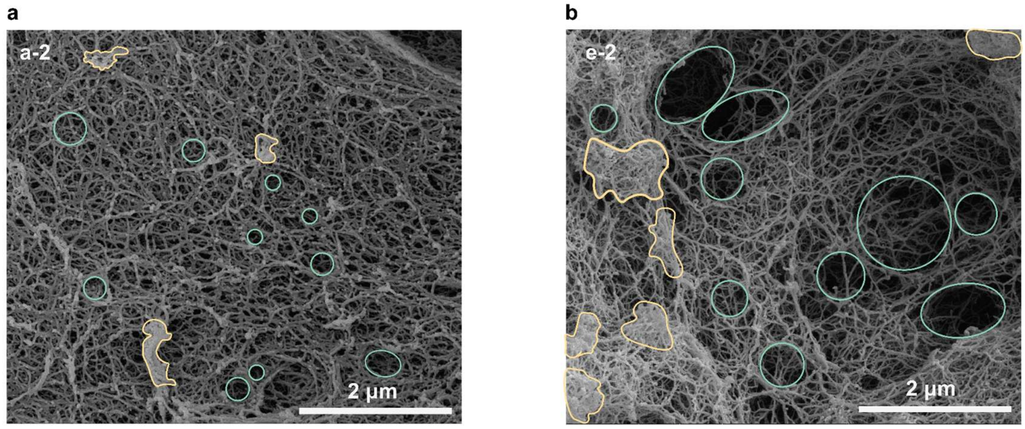

Figure S6. SEM images of PHT aerogels of DML 100 and DMF 100 in combination with the manual identification of complementery pores (green) and polymer clusters (yellow). The images were selected from Figure S2 with 50,000 magnification: a) a-2 and b) e-2.

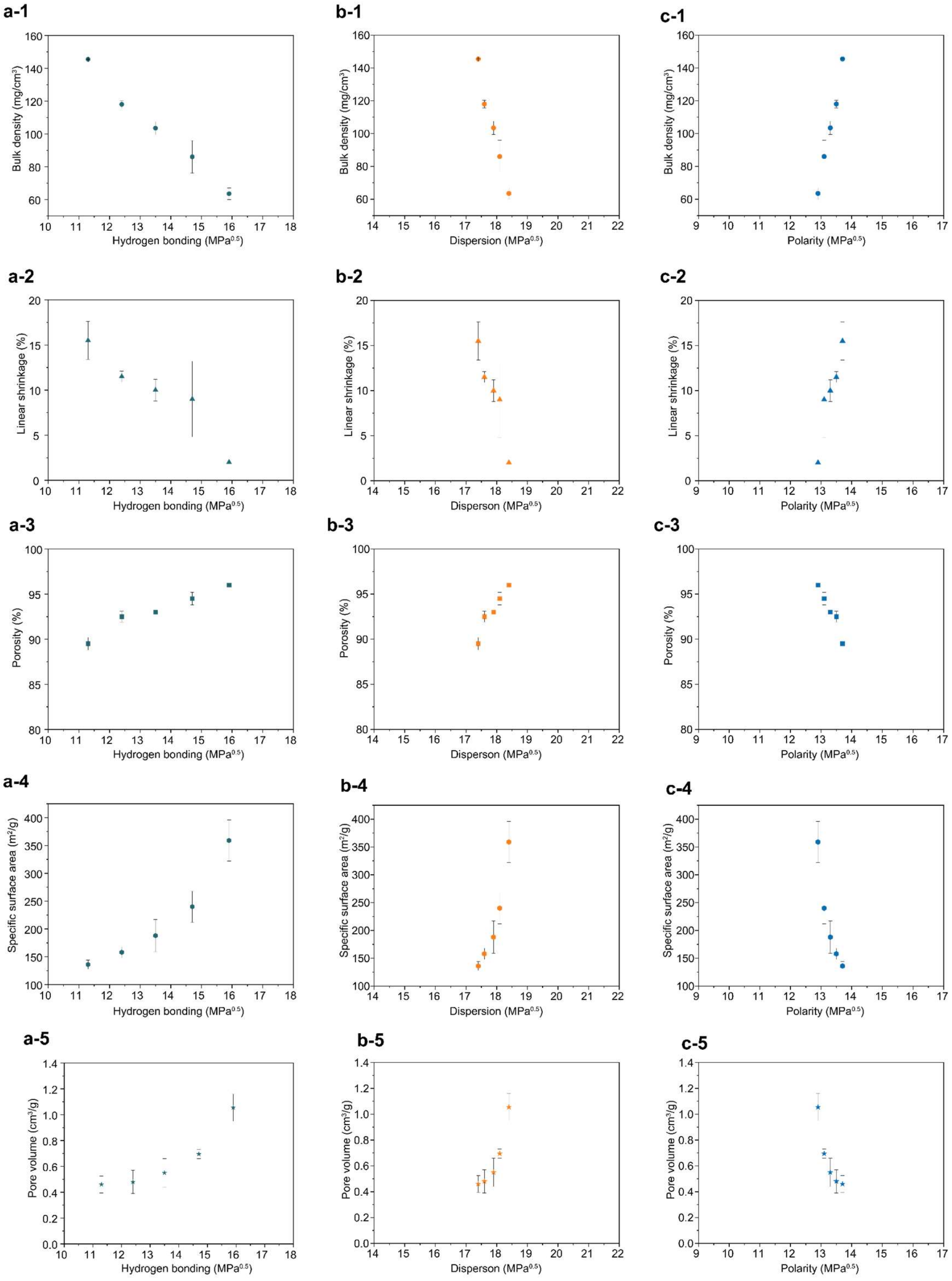

Figure S7. Aerogel-specific properties of PHT aerogels, including, bulk density (mg/cm^3), linear shrinkage (%), porosity (%), specific surface area (m^2/g) and pore volume (cm^3/g)) as functions of the Hansen solubility parameters. Data are plotted for five series of solvent blends (DML 100, DML 75, DML 50, DML 25, and DMF 100) prepared based on **Table S3**, with δ_H = 11.3−15.9 MPa$^{1/2}$ (green, a), δ_D = 17.4−18.4 (orange, b), and δ_P = 12.9−13.7 MPa$^{1/2}$ (blue, c).

4. Closed-loop recycling of PHT aerogel and solvents

Table S6. Recovery yields of the chemical from closed-loop recycling

Name	Recovery yield [%]
BABS	77
DML	83
Ethanol	94

Table S7. Material properties of recycled PHT-1

Recycled PHT-1	Value
Bulk density ρ_b [mgcm^{-3}]	55
Linear shrinkage [%][a)]	2
Skeletal density ρ_s [gcm^{-3}]	1.28
Porosity Π [%][b)]	95
Specific surface area [m^{2}g^{-1}]	396.2
Pore volume [cm^{3}g^{-1}]	0.90
Thermal conductivity [mWm^{-1}K^{-1}]	17.6 ± 0.06
Decomposition temperature at 5% weight loss $T_{d5\%}$ [°C]	258
Decomposition temperature at 30% weight loss $T_{d30\%}$ [°C]	301
Char yield at 793 °C [%]	21.7

[a)]Linear shrinkage was calculated based on the diameter change of the sample; [b)]Porosity was calculated via equation: $\Pi=(1-\rho_b/\rho_s)\times 100\%$.

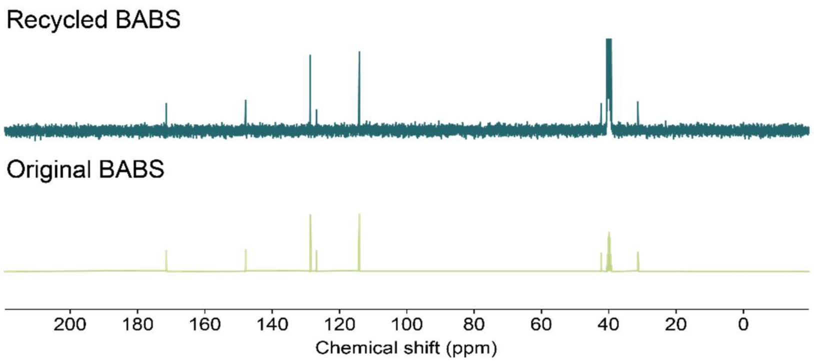

Figure S8. ^{13}C NMR spectra of the original and recycled BABS (100 MHz, 25 °C, DMSO-d_6).

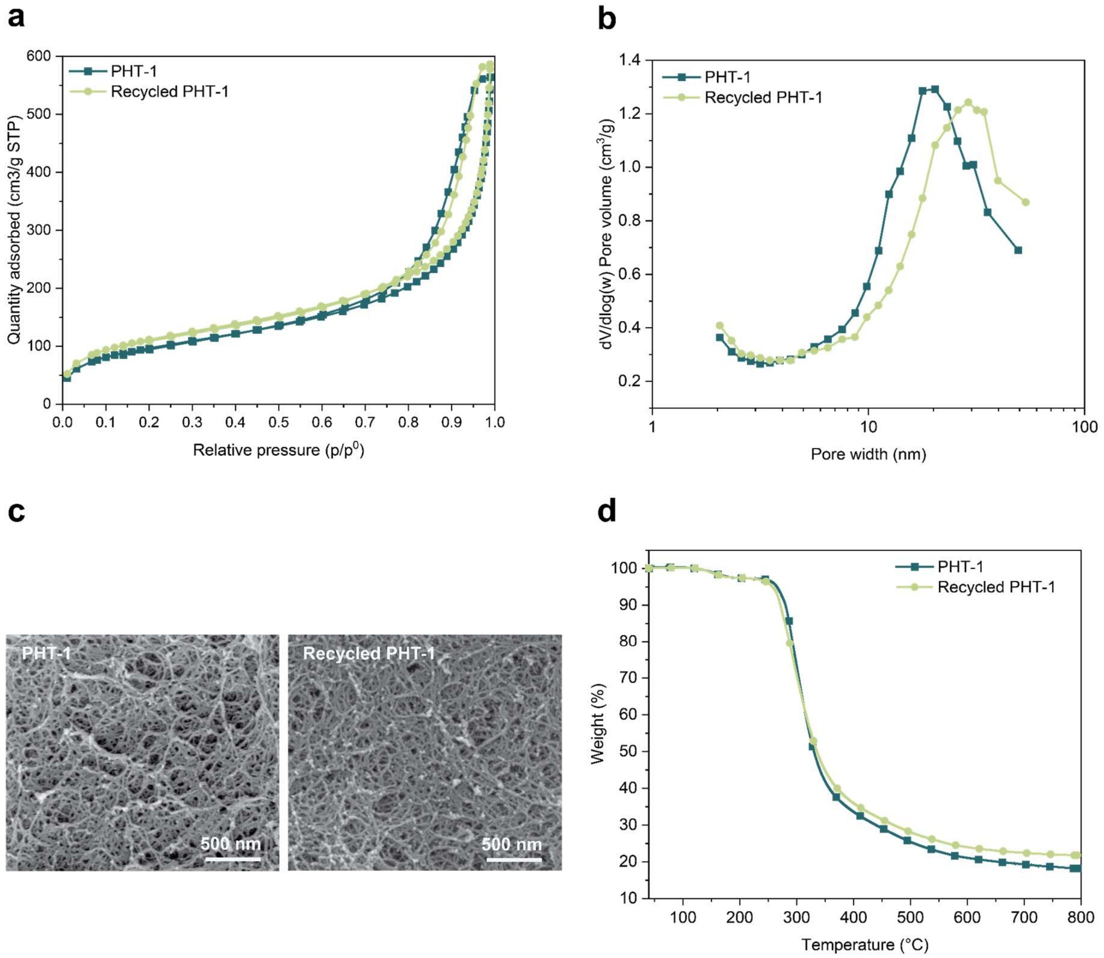

Figure S9. a) Nitrogen physisorption isotherms of original and recycled PHT-1 at 77 K. b) Pore size distribution of original and recycled PHT-1. c) SEM images of original and recycled PHT-1. d) TGA curves of PHT aerogels ramping from 40 °C to 793 °C with ramp rate of 10 °C/min.

5. Supplementary figures

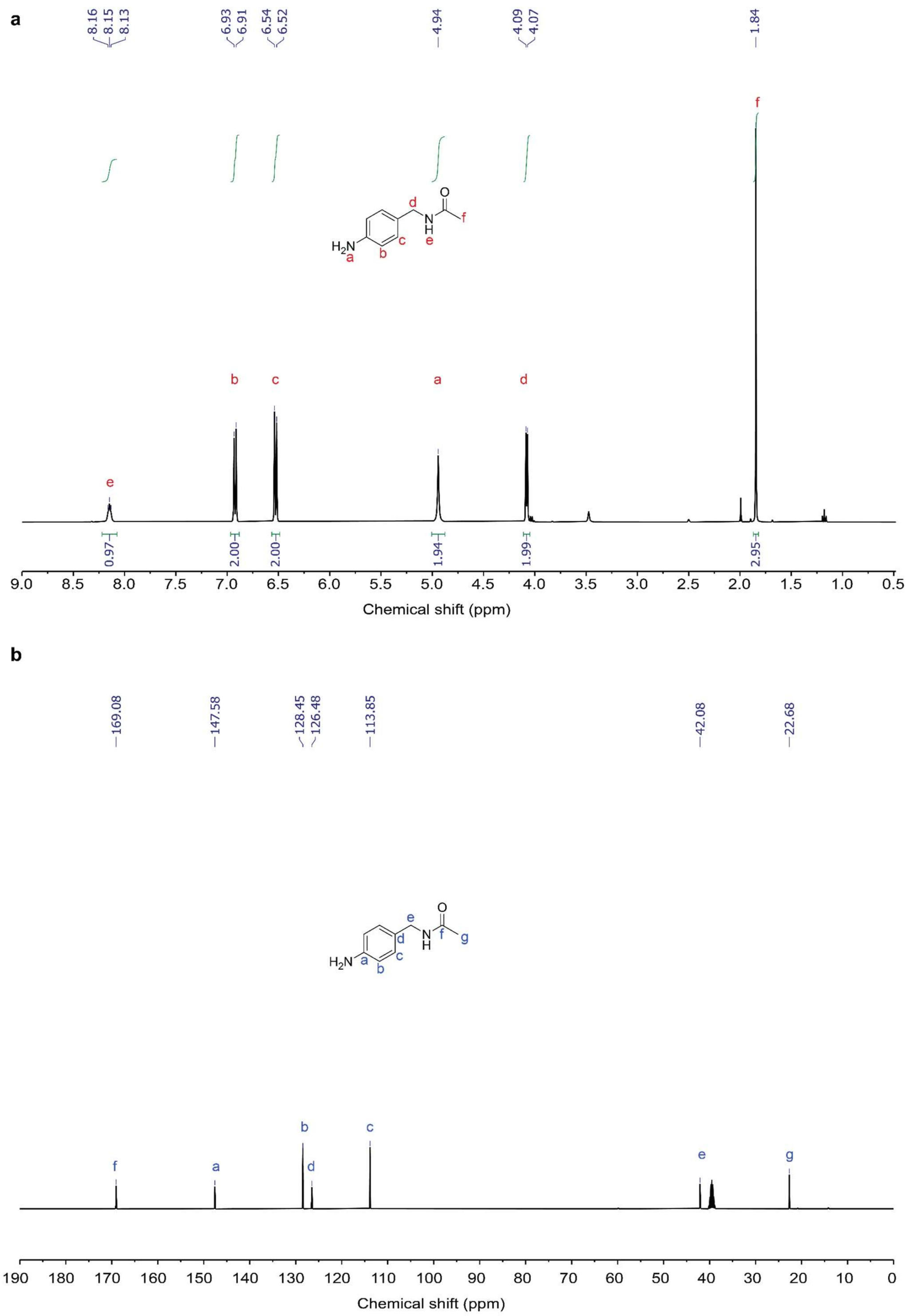

Figure S10. a) ^{1}H NMR (400 MHz, 25 °C), b) ^{13}C NMR (100 MHz, 25 °C) spectra of *N*-(4-aminobenzyl)acetamide in DMSO-d_6 as solvent.

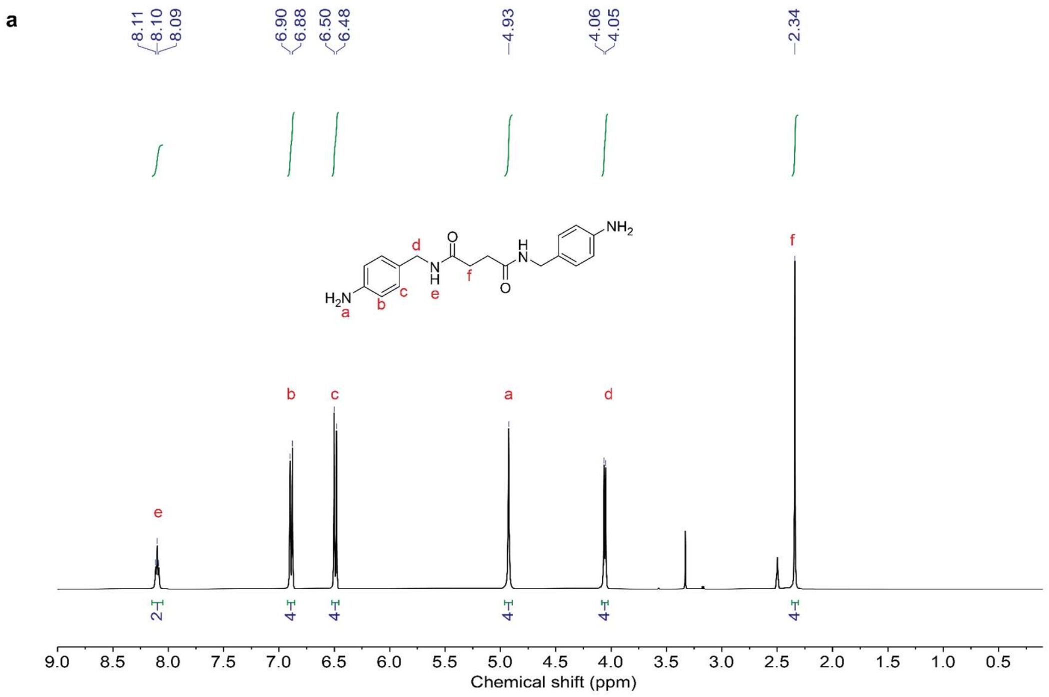

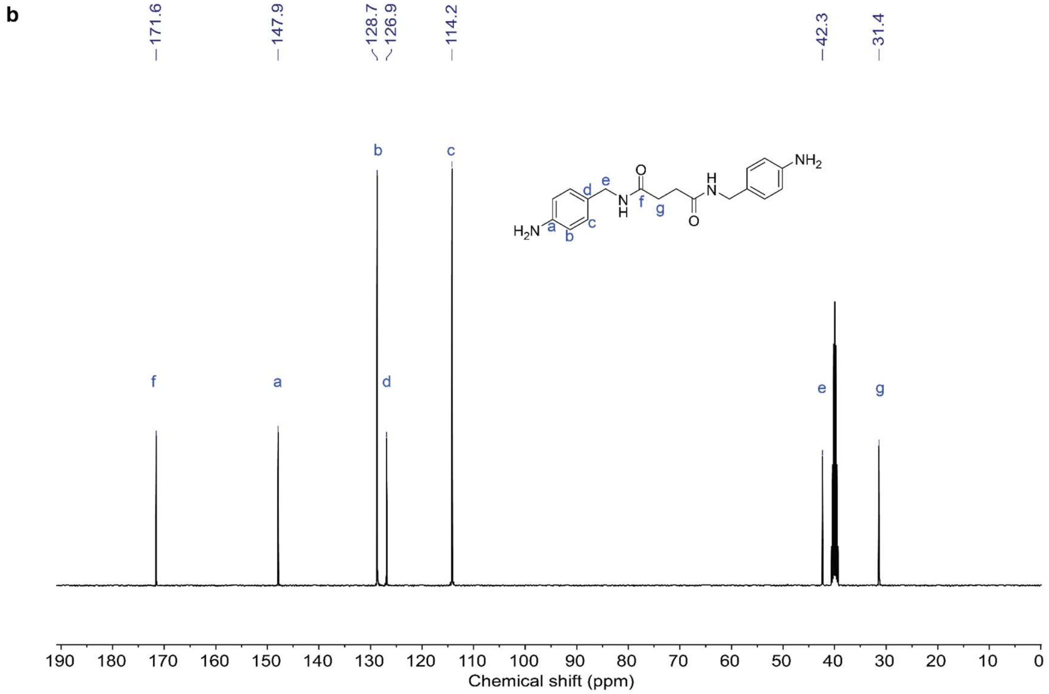

Figure S11. a) ^{1}H NMR (400 MHz, 25 °C), b) ^{13}C NMR (100 MHz, 25 °C) spectra of BABS in DMSO-d_6 as solvent. .

6. References

[1] H. Morimoto, R. Fujiwara, Y. Shimizu, K. Morisaki, T. Ohshima, *Org. Lett.* **2014**, *16*, 2018–2021.

[2] J. K. Lee, G. L. Gould, W. Rhine, *J. Sol-Gel Sci. Technol.* **2009**, *49*, 209.

[3] A. M. Saeed, C. A. Wisner, S. Donthula, H. Majedi Far, C. Sotiriou-Leventis, N. Leventis, *Chem. Mater.* **2016**, *28*, 4867.

[4] N. Leventis, C. Chidambareswarapattar, A. Bang, C. Sotiriou-Leventis, *ACS Appl. Mater. Interfaces* **2014**, *6*, 6872.

[5] T. Taghvaee, S. Donthula, P. M. Rewatkar, H. Majedi Far, C. Sotiriou-Leventis, N. Leventis, *ACS Nano* **2019**, *13*, 3677.

[6] N. Diascorn, S. Calas, H. Sallée, P. Achard, A. Rigacci, *J. Supercrit. Fluids* **2015**, *106*, 76.

[7] R. Trifu, G. Gould, S. White, *MRS Adv.* **2017**, *325*, 1.

[8] B. Merillas, F. Villafañe, M. Á. Rodríguez-Pérez, *Nanomaterials* **2022**, *12*, DOI 10.3390/nano12142409.

[9] J. Tian, Y. Yang, T. Xue, G. Chao, W. Fan, T. Liu, *J. Mater. Sci. Technol.* **2022**, *105*, 194.

[10] Z. Ma, T. Xue, Q. Wali, Y. E. Miao, W. Fan, T. Liu, *Compos. Commun.* **2023**, *39*, 101528.

[11] O. A. Tafreshi, S. Ghaffari-Mosanenzadeh, S. Karamikamkar, Z. Saadatnia, S. Kiddell, C. B. Park, H. E. Naguib, *J. Mater. Chem. C* **2022**, *10*, 5088.

[12] K. Yao, S. Jiang, S. Li, C. Zhang, H. Hou, *Compos. Commun.* **2023**, *38*, 101503.

[13] M. Alshrah, M. P. Tran, P. Gong, H. E. Naguib, C. B. Park, *J. Colloid Interface Sci.* **2017**, *485*, 65.

[14] M. Schwan, R. Tannert, L. Ratke, *J. Supercrit. Fluids* **2016**, *107*, 201.

[15] H. Ren, J. Zhu, Y. Bi, Y. Xu, L. Zhang, *J. Porous Mater.* **2017**, *24*, 1165.

[16] J. C. Williams, M. A. B. Meador, L. McCorkle, C. Mueller, N. Wilmoth, *Chem. Mater.* **2014**, *26*, 4163.

[17] C. M. Hansen, *Hansen Solubility Parameters: A User's Handbook*, *Second Edition*, CRC Press, **2017**.

[18] O. Gronwald, M. Weber, *J. Appl. Polym. Sci.* **2020**, *137*, 1–12.
